# Oxygen Saturation Curve Analysis in 2298 Hypoxia Awareness Training Tests of Military Aircrew Members in a Hypobaric Chamber

**DOI:** 10.3390/s24134168

**Published:** 2024-06-27

**Authors:** Manuel Alvear-Catalán, Claudio Montiglio, Danilo Aravena-Nazif, Ginés Viscor, Oscar F. Araneda

**Affiliations:** 1 Centro de Medicina Aeroespacial (CMAE), Fuerza Aérea de Chile, Santiago 7550000, Chile; manuel.alvearcatalan@fach.mil.cl (M.A.-C.); cmontiglio@fach.mil.cl (C.M.); daravena@udd.cl (D.A.-N.); 2 Secció de Fisiologia, Departament de Biologia Cel·lular, Fisiologia i Immunologia, Facultat de Biologia, Universitat de Barcelona, 08028 Barcelona, Spain; gviscor@ub.edu; 3 Facultad de Medicina, Clínica Alemana Universidad del Desarrollo, Santiago 7610315, Chile; 4 Integrative Laboratory of Biomechanics and Physiology of Effort, (LIBFE), School of Kinesiology, Faculty of Medicine, Universidad de los Andes, Santiago 7620001, Chile

**Keywords:** acute hypobaric hypoxia, aircrews, hypoxia awareness training, peripheral oxygen saturation, slope of arterial oxygen saturation curve

## Abstract

We aim to provide reference values for military aircrews participating in hypoxia awareness training (HAT). We describe several parameters with potential biomedical interest based on selected segments and slopes of the changes in oxygen saturation (SatO_2_) during a standard HAT. A retrospective analysis of 2298 records of the SatO_2_ curve was performed, including 1526 military men aged 30.48 ± 6.47 years during HAT in a hypobaric chamber. HAT consisted of pre-oxygenation at 100% and an ascent to 7620 m, followed by O_2_ disconnection starting the phase of descent of SatO_2_ until reaching the time of useful consciousness (TUC), and finally reconnection to 100% O_2_ in the recovery phase. Using an ad hoc computational procedure, the time taken to reach several defined critical values was computed. These key parameters were the time until desaturation of 97% and 90% (hypoxia) after oxygen mask disconnection (D97/D90) and reconnection (R97/R90) phases, the time of desaturation (TUC-D97) and hypoxia (TUC-D90) during disconnection, the total time in desaturation (L97) or hypoxia (L90), and the slopes of SatO_2_ drop (SDSAT97 and SDSAT90) and recovery (SRSAT97). The mean of the quartiles according to TUC were compared by ANOVA. The correlations between the different parameters were studied using Pearson’s test and the effect size was estimated with ω^2^. Potentially useful parameters for the HAT study were those with statistical significance (*p* < 0.05) and a large effect size. D97, D90, R97, and R90 showed significant differences with small effect sizes, while TUC-D97, TUC-D90, L97, L90, and SDSAT97 showed significant differences and large effect sizes. SDSAT97 correlated with TUC (R = 0.79), TUC-D97 (R = 0.81), and TUC-D90 (R = 0.81). In conclusion, several parameters of the SatO_2_ curve are useful for the study and monitoring of HAT. The SDSAT97 measured during the test can estimate the TUC and thus contribute to taking measures to characterize and protect the aircrew members.

## 1. Introduction

The first report on the effects of acute hypobaric hypoxia associated with human flight dates from a meteorological scientific expedition led by James Glaisher, who ascended with Henry Coxwell in a hot air balloon to 8839 m. In his article published in the Lancet in 1862, Glaisher describes the appearance of tachycardia, respiratory distress, paralysis of the limbs, and visual disturbances resulting from ascent [1,2]. From then until today, the great development of aviation for military and commercial purposes has been the engine for studying physiological changes and generating technological strategies to generate safe conditions in flights [3]. In this way, innovations have been developed in aircraft monitoring and design to reduce accidents and incidents due to hypoxia in flight. Thus, in 1913, in the most important milestone of its time, supplemental oxygen delivery systems were incorporated into aircraft [4], and later in 1921, cabin pressurization was installed [5]. Another important landmark occurred in 1942, when the U.S. Navy implemented the first training program of simulated flight in a hypobaric chamber. Its objective was to instruct aviators on the physiological effects of changes in atmospheric pressure, familiarize them with hypoxia-induced symptomatology, and train skills to prevent sudden incapacitation during flight. These initiatives originated what has subsequently been termed “hypoxia awareness training” (HAT) [6,7], a standardized test that is currently replicated by most of the world’s air forces, becoming the most effective tool to prevent catastrophic consequences derived from in-flight hypoxia events [8,9].

HAT is performed either in a hypobaric chamber or by using normobaric devices, simulating flight conditions under strict safety regulations, using a protocol relatively standardized by most of the world’s military air forces [10,11]. The maximum exposure height during the test (reached in minutes) fluctuates between 3810 m and 13,106 m, which causes the appearance of symptoms such as headache, paresthesia, cognitive deficits, dizziness, and hot flashes [12,13]. Thus, the protocol developed focuses on the recognition of these symptoms and, for this reason, is regularly carried out by crews for periods between three and five years [9]. During HAT, in addition to the onset of symptoms, reflecting the drop in partial pressure of oxygen, a fall in SatO_2_ is observed, which can reach close to 60% according to Yoneda et al. [14]. In addition, there is an increase in heart rate due to a decrease in parasympathetic tone and a relative increase in sympathetic tone [15]. Based on these physiological changes and to minimize the risks during training, many military-grade hypobaric chambers monitor their subjects through pulse oximetry to ensure the timely administration of oxygen, either by the subject being evaluated or by the medical personnel conducting the test acting to prevent syncope from occurring due to hypoxia [16]. Previously, using this monitoring method, some studies have been published partially describing the pulse oximetry changes during HAT; however, these reports involved a low number of participants (n < 50), which makes it difficult to adequately describe the behavior of the physiological parameters of interest [17,18]. Despite this, some segments of possible interest have been proposed, both on the biological events themselves and on the search for the description of their relationships with the rest of the elements that make up the test, such as the appearance of symptoms or the time of useful consciousness (TUC). The interest of the study of the SatO_2_ curve is to contribute both to the search for predictors/diagnoses of human responses in these conditions and to contribute to improving the safety of the evaluated people during the test [19,20]. To a large extent, the detailed study of the pulse oximetry curve has not been standardized because this type of monitoring is not used by all evaluation teams, and also because its study requires the simultaneous synchronized acquisition and analysis of a large amount of data, thus making it mandatory to create and develop a signal acquisition and analysis system specifically designed for these purposes. Focusing on this problem, our group recently published the development of an “ad hoc” open-source computational procedure for the evaluation of SatO_2_ and heart rate during HAT that allows the simultaneous analysis of a large volume of data [21]. Into this tool, we incorporated the calculations of different curve parameters (segments/slopes) to which we have theoretically assigned a potential biological and medical interest. In this paper, we show the analysis of eleven parameters obtained from SatO_2_, reporting the expected values in military aircrew and the associations between the variables proposed with the TUC in 2298 HAT tests carried out in the hypobaric chamber of the Chilean Air Force between 2010 and 2018.

## 2. Materials and Methods

Participants: A total of 2298 records were obtained from 1526 healthy male subjects aged 30.48 ± 6.47 years, non-acclimatized to high altitudes. All the studied subjects were specialized military personnel in the roles of airplane or helicopter pilot, aircrew member, or paratrooper of the Armed Forces of the Republic of Chile. Subjects with current aeromedical certification who participated either for the first time or within three years of their last hypoxic training were designated to participate in HAT as required by institutional regulations between 2010 and 2018. The records obtained during the test were anonymized by a member of the research team and identified with a code. The use of this database for research purposes was approved by the research ethics committee of the Chilean Air Force Hospital (Santiago de Chile) under number 102310.

HAT Protocol: Before the flight simulation, the subject’s tubal function was assessed by acoustic impedance, in addition to participating in a theoretical instruction on the test. Once inside the chamber, an ascent was made to 2438 m (8000 ft), verifying in situ the ability to equalize the pressures of the middle ear, nasal, and paranasal sinuses. Subsequently, at this altitude, 30 min of denitrogenation were performed to avoid decompression alterations using an aviation mask that provided them with 100% oxygen. At the end of this time, the stage called “diurnal flight” was carried out, in which the pressure inside the chamber was gradually decreased, simulating an ascent at a speed of 914 m/min (2000 ft/min) to 7620 m (25,000 ft) in 8 min. Once this height was reached, the evaluated subjects were instructed to remove the aviation mask and initiate the hypoxia test to carry out the recognition of the symptoms associated with this environmental condition. The end of this phase occurred, by protocol, for the following reasons: (a) on the part of the trainees themselves, (b) by the advice of the medical team, (c) when some degree of compromise of consciousness was detected, (d) because of a decrease in SatO_2_ below 65% [16,22], or (e) when five minutes had elapsed from the start of the test, which corresponds to the maximum time established. Once any of these criteria were met, the evaluates were reconnected to the mask delivering 100% O_2_ added to a positive pressure equivalent to 10 to 15 cm H_2_O until they achieved a level of SatO_2_ of at least 95%; the positive pressure was then removed, but 100% O_2_ supply continued. Subsequently, the effects on visual acuity were evaluated in nocturnal conditions at an altitude of 5486 m (18,000 ft) in conditions of low chamber illumination, a period in which a hypoxia protocol analogous to daytime flight was also performed. The hypobaric chamber then decreased the simulated altitude at a rate of 914 m per minute until it equaled the barometric pressure corresponding to its location level (709 m/2326 ft).

Equipment and recording system: The training was carried out in the hypobaric chamber of the Centro de Medicina Aeroespacial (CMAE) of the Chilean Air Force located in the commune of Las Condes, Santiago de Chile. The chamber corresponds to the M-10 model (Environmental Tectonics Corporation, Southampton, PA, USA), with ten seats for training personnel and two places for evaluators. The facility is equipped with an automated flight control system and has a system for monitoring, acquiring, and storing SatO_2_ data measured with a pulse oximeter Ipod 3211 (Nonin, Plymouth, MN, USA), which has a sampling rate range declared by the manufacturer between 18 to 300 pulses per minute.

**Figure 1:**
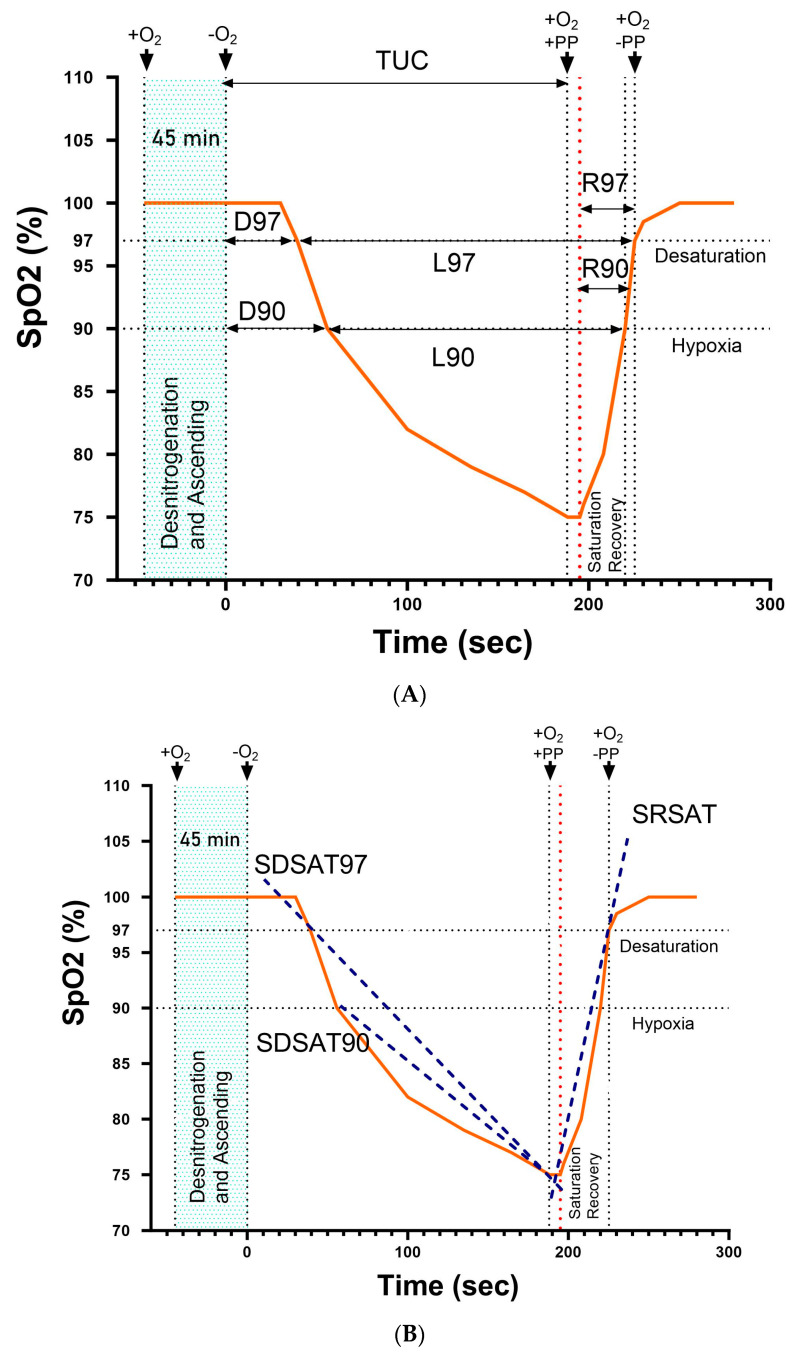
Segments (**A**) and slopes (**B**) of the SatO_2_ curve (colored line) during HAT in the hypobaric chamber. TUC = time of useful consciousness; D97 = desaturation delay; D90 = hypoxia delay; L97 = desaturation time; L90 = hypoxia time; R90 = hypoxia recovery time; R97 = desaturation recovery time; +O_2_/−O_2_ = 100% oxygen mask supply status (on/off); PP = positive pressure (10–15 cm H_2_O); SDSAT97 = desaturation phase slope; SDSAT90 = hypoxia phase slope; and SRSAT97 = recovery phase slope.

**Figure 2:**
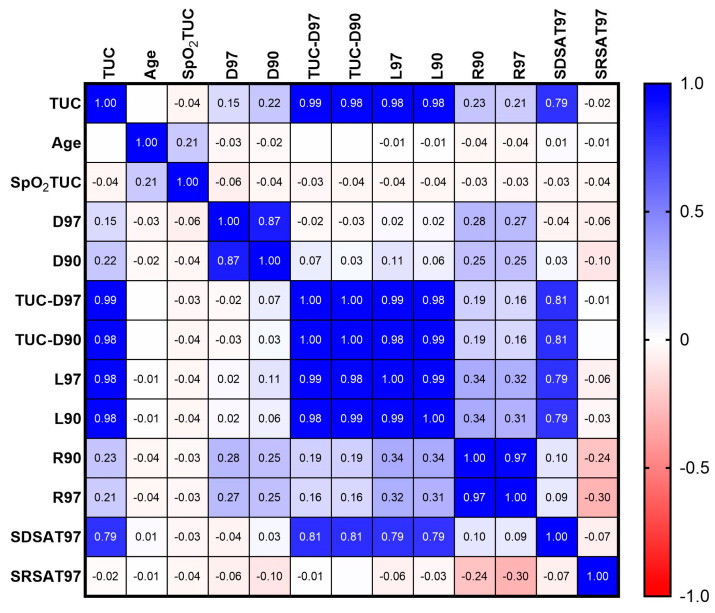
Pearson’s correlation coefficients between the parameters obtained from the SatO_2_ curve during HAT in the hypobaric chamber in 2298 individual records. TUC = time of useful consciousness; SpO_2_TUC = SatO_2_ at TUC; TUC-D97 = desaturation time from disconnection to O_2_ supply; TUC-D90 = hypoxia time during disconnection to the O_2_ mask; D97 = desaturation delay; D90 = hypoxia delay; L97 = desaturation time; L90 = hypoxia time; R90 = hypoxia recovery time; R97 = desaturation recovery time; SDSAT97 = desaturation phase slope; and SRSAT97 = recovery phase slope. Empty cells for R have extremely low values.

Data processing: The records obtained during the HAT allowed us to generate a database of 569 flights, from which a sample of 2298 individual records was extracted, with a total of 33.6% of the records obtained from subjects measured on more than one occasion. Data were acquired individually, storing test time, simulated height, SatO_2_, heart rate, and oxygen mask status (off/on), as well as age and sex. Each flight was processed to find the time intervals corresponding to ascent, simulated maximum altitude, and descent. The individual data were then pre-processed, removing from the final analysis records with missing data, sensor noise, and a small number of outliers (n = 55). Subsequently, the records were analyzed using a platform, designed by our research group, called “Physiological Analysis of Hypoxic Simulated Flight” [21], developed with the Igor Pro 8.01 software platform (WaveMetrics, Portland, OR, EE. UU.). This platform has a procedure designed to detect phenomena typical of the SatO_2_ curve during HAT considered by our group as potentially of physiological relevance (see a graphical description of the proposed parameters in Figure 1 and Figure 2). To identify these phenomena, in part, we based our parameters on the report by Malle et al. [19], who proposed three phases of curve study (see description below), the rest of the parameters being established by our group. The description of these parameters is shown in Figure 1A, B and detailed below:

(a) TUC: corresponds to the time that elapses between the disconnection and reconnection of the oxygen mask during the test [23], with a maximum time, per protocol, of five minutes. This measurement corresponds to a standard parameter in HAT tests.
(b) SpO_2_TUC: oxygen saturation at the time of TUC.
(c) Desaturation delay (D97): corresponds to the time until SatO_2_ reaches a value of 97% after the 100% O_2_ mask is off. This parameter is similar to the “desaturation-delay phase” proposed by Malle et al. [19]. However, these authors used a limit value of 98%, while in our case we decreased it to 97% to reduce the frequent effect of oscillations.
(d) Hypoxia delay (D90): corresponds to the time until SatO_2_ reaches a 90% value after the 100% O_2_ mask is off.
(e) Desaturation Time (L97): corresponds to the time in which those evaluated show SatO_2_ values below 97%.
(f) Hypoxia Time (L90): corresponds to the time in which those evaluated show SatO_2_ values below 90%.
(g) Desaturation time during disconnection to O_2_ supply (TUC-D97): is the time during which SatO_2_ decreases from disconnection to reconnection. It is analogous to the “desaturation phase” of Malle et al. [19]. It is calculated as the difference of TUC minus D97.
(h) Hypoxia time during disconnection to the O_2_ mask (TUC-D90): corresponds to the time when SatO_2_ decreases from 90% until reconnection to the 100% oxygen mask. For its calculation, TUC minus D90 was subtracted.
(i) Desaturation Recovery Time (R97): the time elapsed, once the oxygen mask has been reattached, between the onset of the increase in SatO_2_ to reach 97%. It is analogous to the “resaturation delay” phase of Malle et al. [19]; however, these authors measure this segment from the moment the oxygen mask is reattached.
(j) Hypoxia recovery time (R90): the time elapsed, once the oxygen mask has been reconnected, between the onset of the increase in SatO2 to 90%.
(k) Desaturation phase slope (SDSAT97): corresponds to the slope of a line drawn between the value of 97% and the point of reconnection to oxygen on the SatO_2_ curve.
(l) Hypoxia phase slope (SDSAT90): corresponds to the slope of a line drawn between the value of 90% and the point of reconnection to oxygen on the SatO_2_ curve. This parameter is not calculated automatically by the computational procedure. However, it is possible to obtain it manually, for which a part (n = 100) of the total sample was randomly selected.
(m) Recovery phase slope (SRSAT97): calculated as the slope of the line drawn between the starting point of the rise in SatO_2_ after the application of oxygen and 97% saturation.

Data treatment: In the present report, we exclusively analyze the so-called diurnal flight phase (7620 m) of the total records. In this sample, we use the quartile division of TUC as a strategy to find potential parameters of interest, comparing the averages and looking for potential correlations between the different parameters. Since the desaturation slope (SDSAT97) showed a strong correlation with the parameters TUC, TUC-97, TUC-90, L97, and L90 (see results), we considered it essential to investigate if these relationships were also maintained with the hypoxia phase slope (SDSAT90). Due to this slope not being automatically generated by the computer program, we randomly selected 25 records in each quartile (n = 100) to obtain it manually and check the relationships between SDSAT90 with the rest of the variables and with SDSAT97.

Statistical analysis: The normality of each parameter was determined through the graphical evaluation of its distribution, arbitrarily establishing as limits an absolute obliquity value ≤ 2 or an absolute kurtosis value ≤ 4, as suggested for a sample size higher than 300 observations [24,25]. For the data analysis, the whole dataset was divided into quartiles based on the TUC, since this was the most relevant parameter obtained during the physiological test. As a strategy to find parameters of interest from the SatO_2_ curve, those quartiles were compared through the application of a one-way ANOVA test and the calculation of the effect size was conducted through the determination of ω^2^. Measures that were shown to be significant in the test with a large effect size were considered to be of interest. The criteria used to interpret the effect size were 0.01, small; 0.06, medium; and 0.14 large, according to Kirk [26]. The correlations between the different segments and slopes of the SatO_2_ curve were determined by calculating Pearson’s correlation coefficient. For the interpretation of the R values, the criteria proposed by Schober et al. [27] were applied. Thus, values below 0.10 were considered very weak, between 0.10 and 0.39 weak, between 0.40 and 0.69 moderate, between 0.70 and 0.89 strong, and greater or equal to 0.90 very strong. A statistical significance level of *p* < 0.05 was established. The GraphPad Prism package version 9.0.0 (GraphPad Software, San Diego, CA, USA) was used for the statistical analysis.

## 3. Results

When comparing with the ANOVA test the averages of the different parameters, grouped according to the TUC quartiles, we found no differences concerning age (average value close to 30 years), nor in the minimum SatO_2_ registered during the test (average value close to 72%). Regarding the parameters of the SatO_2_ curve, significant differences were found, but with a small effect size, for D97, D90, R97, and R90. In addition, significant differences were evidenced with a large effect size for TUC, TUC-97, TUC-90, L97, and L90. Regarding slopes, we found significant differences for SDSAT97 and SRSAT97, with a small effect size for the recovery slope and a large one for the desaturation slope (Table 1).

Regarding the observed relationships between the parameters (Pearson’s correlation coefficient), we found a weak direct correlation between age and minimum SatO_2_ during the test. D97 and D90 showed a strong correlation between them, while both correlated weakly with R90 and R97. Further, R90 and R97 showed a positive weak correlation with TUC, TUC-D90, TUC-D97, D90, D97, L90, and L97, whereas a weak negative relationship with SRSAT97, this being the only association evidenced for this latter parameter. Regarding TUC, weak direct correlations were found with D97, D90, R97, and R90, while there were very strong correlations with TUC-97, TUC-90, L97, and L90 in addition to showing a strong correlation with SDSAT97. The latter parameter was strongly correlated with TUC-97, TUC-90, L97, and L90. As noted above, the procedure designed for the analysis does not allow for the automatic calculation of the value of the SDSAT90, but it can be calculated manually. Thus, in this subsample (n = 100), the quartiles of the same parameters calculated in the total sample were obtained. For reference, we found TUC values (mean and standard deviation per quartile from Q1 to Q4) of 103.9 ± 21.20, 169.0 ± 10.58, 212.6 ± 15.68, and 264.6 ± 18.18 s; (F = 407.5, *p* < 0.0001, and ω2 = 0.924). The SDSAT97 values were −0.177 ± 0.11, −0.172 ± 0.034, −0.128 ± 0.033, and −0.09 ± 0.023; (F = 8.02, *p* < 0.0001, and ω^2^ = 0.107) from Q1 one to Q4, respectively. In addition, SDSAT90 showed the following values, also ordered from Q1 to Q4: −0.253 ± 0.103, −0.144 ± 0.038, −0.106 ± 0.030, and −0.075 ± 0.020; (F = 44.73, *p* < 0.0001, and ω^2^ = 0.5673), respectively. Regarding the correlations for SDSAT90, we found as a relevant result a close correlation between the values of SDSAT97 (R = 0.98 and *p* < 0.0001) and TUC (R = 0.80 and *p* < 0.0001), in addition to trends and correlation coefficients similar to those found in the total sample for SDSAT97, as reported in the correlation matrix of Figure 2.

## 4. Discussion

Military aircrews can experience acute hypoxia, increasing the possibility of accidents and putting their lives and the operations of their duties at risk. For this reason, for several decades, HAT that simulates this emergency has been implemented. Thus, throughout these years of modern aeronautical development, thousands of air crews have participated in this training all over the world. Regarding this, since its primary objective is the recognition of symptoms induced by hypoxia, the measurement of peripheral oxygenation is not mandatory, even though, in most training centers, this monitoring is carried out. This report, to our knowledge, is the first time such a large series of analyses of the SatO_2_ curve in HAT exposure has been published. In addition, due to the use of a computational procedure that allows the processing of a large amount of data, we can propose segments of the curve of interest that can contribute knowledge both to hypoxia research and to improving the safety of the test. Thus, as the most relevant result, we have found that there is no relationship between the useful time of consciousness and the SatO_2_ at the TUC reached during the test, nor with the age of those evaluated, but there are close correlations with the slope of fall of SatO_2_ from the value 97 and 90 until reaching the end of the hypoxia phase.

In our group, the first step to study the segments and slopes of the SatO_2_ curve during HAT originated from observing its morphology in the tests carried out in our center. In addition, as previously mentioned, this was added to the initial description of some segments by other research groups. The first parameters proposed correspond to the times it takes those evaluated to reach, from the withdrawal of 100% oxygen, 97% and 90% of SatO_2_, which we have set as the limit of “desaturation” and “hypoxia”, respectively. We have worked with these two parameters because, during the O_2_ disconnection phase, SatO_2_ can undergo oscillations without changing its downward trend, which potentially has a greater effect on the value of 97%. In addition, we have calculated them to determine the time during which those evaluated continue to decrease their SatO_2_ to the TUC, calculated as TUC-D97 and TUC-D90, respectively. Regarding the physiological significance of these segments analyzed at this extreme altitude in the so-called daytime flight phase (7620 m), it is likely that their values are mainly influenced by the magnitude of ventilation, since, if this parameter increases, the greater the alveolar air exchange with a low partial pressure of ambient O_2_ (approximately 59 mmHg), generating an accelerated drop in saturation. This makes the relationship between ventilation and blood oxygenation, at this altitude, a paradoxical phenomenon if we look at the relationship between these parameters both at sea level or at lower altitudes than the one studied in this work [28]. Second, it is also likely to affect the magnitude of pulmonary blood flow [29,30] as a reflection of overall cardiovascular activity; however, we think that this factor plays a very secondary role during HAT, since those evaluated remain at rest during the exam. Finally, it is also possible that there is an influence both by previous hyperoxia [31] and by blood factors that are not yet elucidated. On the other hand, when comparing the quartiles, we observe that the values of D97 and D90 increase with the TUC, which can be corroborated by the positive correlation (although weak) with the latter parameter. When calculating the fraction of the TUC represented by D97 and D90, we find a drop in this index with approximate values of 0.33, 0.23, 0.19, and 0.14 for D97 from quartile one to fourth, respectively, for the full sample. These findings point to the fact that those evaluated who fall into desaturation or hypoxia at an accelerated rate will most likely have a decreased TUC; however, we believe that the ability to predict TUC by these indicators is limited, because the effect size calculated between the quartiles was small (Table 1).

In the last phase of the test, once the criteria for reconnection to oxygen have been executed, it is not uncommon to observe that SatO_2_ continues to fall for a few seconds (as seen in Figure 1); after this, the subject recovers the values of 90 and 97% respectively at an average speed five percent higher than the fall of the parameter when they were disconnected. A probable explanation for this phenomenon is based on the fact that the administration of 100% supplementary oxygen is added to the positive pressure of aviation masks up to 95% of SatO_2_ (see Figure 1), leaving this period, and the parameters determined in it, influenced by more factors involved in the results than the area of pure hypobaric hypoxia of O_2_ disconnection. This is reflected in less variability of recovery times between quartiles compared to their counterpart in the hypoxia phase (TUC). This is good news in practice for the restoration of oxygenation in subjects acutely exposed to severe hypoxia. This acceleration effect of recovery (O_2_ supply restoration + positive pressure) may be partly due to the increase in functional residual capacity by recruiting alveoli that are previously closed, generating an increase in the gas exchange area and thus improving the ventilation/perfusion ratio [32]. Regarding the behavior of the parameters proposed in this phase (R97 and R90), there is a tendency to increase from quartile one to four (Table 1). Observing the SatO_2_ curves, it can be seen that after the instauration of 100% oxygen supply (mask reattached), SatO_2_ continues to fall for a lapse time, and this effect is more pronounced from Q1 to Q4 of TUC. Since the distribution of the slopes is analogous among all the studied subjects, more abrupt drops in SatO_2_ respond more slowly to the administration of O_2_, but once SatO_2_ begins to rise, the recovery is fast, which explains the lower values of R97 and R90 for the first quartiles. Although the trends described are clear, as observed for D97 and D90, because a small effect size is detected, they constitute markers with poor discrimination capacity between the different quartiles. This may also be associated with the fact that the only significant correlations found with these parameters were with the values of D97, D90, and TUC, with which weak correlations were found, which, as a whole, means that its role as an indicator is limited. In summary, both the values proposed immediately after disconnection (D97/D90) and for R97/R90 can only be useful in studies that involve the measurement of those evaluated in protocols that involve pre- and post-measurements or in comparisons of types of hypoxia (i.e., normobaric vs. hypobaric).

Another parameter determined in our report corresponds to the total time that the subjects were under 97 and 90% of SatO_2_, taking into consideration the desaturation and recovery phases in total. This parameter was conceived to be able to contrast its values with other types of parameters that can be measured and crossed when performing HAT, such as cognitive performance tests, determination of saturation in other tissues [22], or cardiovascular or lung function parameters [33]. This indicator can also be useful in determining any blood changes measured before and after hypoxia. Regarding its behavior according to the TUC, we found significant differences with large effect sizes between the quartiles and a strong correlation with the TUC. However, despite this, its role as a TUC predictor is limited because to determine it, the HAT protocol must be completed.

The most interesting result of this report corresponds to the determination of the slopes of the saturation decay curve during HAT. Thus, we found that both the SDSAT97 and the SDSAT90 showed a close correlation with the TUC, the TUC-D97, and the TUC-D90. Furthermore, these findings indicate that these slopes can be useful to predict their values during the daytime flight phase of HAT. In addition, its large effect size, evidenced by comparing the quartiles for these three parameters (see Table 1) and the adjusted correlation in a linear model (see Figure 2), demonstrates that these values do offer the possibility of predicting the TUC, and it is necessary to subsequently search for the model that best explains this relationship. From a practical point of view, our finding will allow, through a programmed function (in development), to monitor these slopes in real-time during the test and to know which of the subjects evaluated simultaneously will reach their TUC first. This will provide the advantage of being able to take measures in advance, thus helping to avoid the occurrence of any adverse event during the test. Regarding the SRSAT97 in the comparison by quartiles according to TUC, a low effect size and only weak correlations with R97/R90 were observed; therefore, its role as a parameter to be considered in the study of the SatO_2_ curve is very limited.

As potential limitations of our work, it should be noted first that the lower SatO_2_ values measured in almost 30% of the sample are near the clinical validation limit (70%) of the devices that determine pulse oximetry [34], although this does not mean that these values correspond to erroneous determinations. To check the influence of these data on the total of our observations, we assessed the correlation between TUC and SDSAT97 in subjects with values lower than 70% of SatO_2_, finding a good fitting (R = 0.80; n = 712) between both parameters. Another limiting aspect is that in our series there are subjects who performed HAT on more than one occasion; however, it is difficult to know what degree of influence this may have on our results until a specific analysis is carried out in this regard. Nonetheless, as preliminary background in our report, we found no relationship between TUC and SDSAT97 concerning the age of the subjects (see Figure 2). Finally, in this database, we do not have segmented information regarding the reason for reconnection to oxygen; however, in our experience most subjects (close to 70%) reconnect to oxygen on their own, while others are stimulated to do so by the medical team for an SatO_2_ < 65%, and the rest reach the total exposure time (see Section 2).

## 5. Conclusions

The analysis of the behavior of SatO_2_ during HAT in a large number of subjects allows us to propose some reference values, not only for internal use but also for other groups that determine the same parameters during a standard HAT test or for researchers interested in the effects of acute exposure to extreme hypoxia. Establishing the values of 90 and 97% of SatO_2_ as arbitrary limits, the proposed segments include both the time in which those evaluated subjects maintain SatO_2_ values above these limits after 100% O_2_ is off (D97/D90), as well as the time it takes to recover once 100% O_2_ supply is restored (R97/R90). According to our analysis, both types of parameters can be used in studies that compare interventions during HAT or to study the different ways of acutely inducing artificial hypoxia that are often used today for different purposes (sports and/or biomedical). The time (L97/L90) below the proposed limit values can be used to study its correlation with physiological changes, blood markers, or cognitive performance, while the slopes of the SatO_2_ decline phase (SDSAT97/SDSAT90), by closely correlating with the TUC, can potentially help with immediate estimation of TUC during the test, allowing preventive actions to benefit the safety of the evaluated subjects.

## Figures and Tables

**Table 1:** Comparison of quartiles of TUC, age, SpO_2_TUC, segments, and slopes of the SatO_2_ curve obtained during HAT in a hypobaric chamber.

	Q1	Q2	Q3	Q4	F	*p*-Value	ω^2^
Sample (n)	583	569	582	564			
TUC (s)	106 ± 21.93	163.6 ± 13.24	210.8 ± 16.24	275.1 ± 17.96	9461	<0.0001	0.9250
Age (y)	30.55 ± 6.66	30.41 ± 6.41	30.45 ± 6.32	30.5 ± 6.39	0.047	0.98	
SpO_2_TUC (%)	72.99 ± 5.27	72.33 ± 5.072	72.7 ± 5.70	72.35 ± 5.33	1.99	0.1125	
D97 (s)	36.66 ± 11.87	39.22 ± 11.03	40.74 ± 9.93	40.51 ± 10.46	17.23	<0.0001	0.020
D90 (s)	52.07 ± 13.53	54.98 ± 12.12	57.42 ± 11.59	59.06 ± 12.0	35.10	<0.0001	0.042
TUC-D97 (s)	69.34 ± 23.13	124.4 ± 16.72	170 ± 19.13	234.6 ± 20.66	6984	<0.0001	0.90
TUC-D90 (s)	53.93 ± 24.09	108.6 ± 17.47	153.3 ± 19.82	216 ± 20.86	6301	<0.0001	0.89
L97 (s)	103.2 ± 25.14	161.8 ± 19.03	208.2 ± 20.42	274.5 ± 22.90	6222	<0.0001	0.890
L90 (s)	81.73 ± 26.1	140.5 ± 19.85	186 ± 20.79	250.3 ± 22.9	5728	<0.0001	0.882
R97 (s)	33.81 ± 9.56	37.36 ± 11.07	38.11 ± 10.94	39.89 ± 11.31	32.62	<0.0001	0.039
R90 (s)	27.73 ± 8.77	31.82 ± 10.52	32.6 ± 10.35	34.17 ± 10.62	42.84	<0.0001	0.051
SDSAT97	−0.29 ± 0.10	−0.17 ± 0.05	−0.12 ± 0.03	−0.085 ± 0.02	1141	<0.0001	0.59
SRSAT97	1.214 ± 0.43	1.306 ± 0.52	1.291 ± 0.53	1.195 ± 0.53	6.80	<0.0001	0.0075

TUC = time of useful consciousness; SpO_2_TUC = SatO_2_ at TUC; TUC-D97 = desaturation time from disconnection to O_2_ supply; TUC-D90 = hypoxia time during disconnection to the O_2_ mask; D97 = desaturation delay; D90 = hypoxia delay; L97 = desaturation time; L90 = hypoxia time; R90 = hypoxia recovery time; R97 = desaturation recovery time; SDSAT97 = desaturation phase slope; and SRSAT97 = recovery phase slope.

## Data Availability

Access to the data is limited by the security policies of the Chilean Air Force. However, the authors can provide some data to researchers upon request.
